# A Portal-Rex Shunt Using Patent Proximal Main Portal Vein as Venous Inflow and Internal Jugular Vein as Conduit

**DOI:** 10.3390/children13020291

**Published:** 2026-02-19

**Authors:** Irene Wen Hui Tu, Yang Yang Lee, Vidyadhar Padmakar Mali

**Affiliations:** Department of Paediatric Surgery, National University Hospital, Singapore 119074, Singapore; yang_yang_lee@nuhs.edu.sg (Y.Y.L.); vidyadhar_mali@nuhs.edu.sg (V.P.M.)

**Keywords:** portal-Rex shunt, extrahepatic portal vein obstruction (EHPVO), modified meso-Rex shunt

## Abstract

**Highlights:**

**What are the main findings?**

- A proximal intact and patent portal vein may be an effective alternative inlet during surgery for a meso-Rex shunt.
- A portal-Rex shunt using an internal jugular vein as a conduit provided good patency and physiological response over a one-year follow-up.

**What are the implications of the main findings?**

- Careful preoperative imaging of the residual splanchnic anatomy in extrahepatic portal vein obstruction (EHPVO) may reveal alternatives for a modification of the meso-Rex shunt.
- The availability of good-calibre intra-abdominal veins may avoid the need for neck exploration and associated morbidity.

**Abstract:**

**Background**: Extrahepatic portal vein obstruction (EHPVO) accounts for more than two thirds of pediatric portal hypertension. Rex shunt is the preferred surgical management, as it restores hepatopetal flow and minimizes or reverses liver dysfunction. **Case Summary**: We report surgical treatment of EHPVO in a 9-year-old girl using portal-Rex shunt with internal jugular vein (IJV) as a conduit and the intact proximal main portal vein instead of the superior mesenteric vein as a venous inlet. Improvement in thrombocytopenia and reduction in splenic size were achieved post-operatively. The portal-Rex shunt remains patent with good hepatopetal flow at one year post-operation. **Conclusions**: The success of a portal-Rex shunt to selectively bypass EHPVO rests upon careful selection of inlet and outlet veins, as well as a conduit with good patency, such as the IJV.

## 1. Introduction

Extrahepatic portal vein obstruction (EHPVO) is one of the most common causes of pediatric portal hypertension, accounting for more than two thirds of portal hypertension in children [1,2]. Children can present with upper gastrointestinal bleeding from gastro-oesophageal varices or symptomatic hypersplenism with thrombocytopenia [3,4]. Surgery is indicated in cases of refractory variceal hemorrhage, encephalopathy, porto-pulmonary syndrome or severe hypersplenism [3]. Surgical treatment options include portal-systemic bypass and the more physiological meso-portal bypass [4]. First described by de Ville in 1992, the meso-Rex shunt is a meso-portal bypass used to treat post-liver transplant portal vein thrombosis [5]. The classic meso-Rex shunt involved using the superior mesenteric vein (SMV) as the venous inflow and connecting it to the intrahepatic left portal vein (LPV) using the internal jugular vein (IJV) as a conduit. Unlike portal-systemic shunts, the meso-Rex shunt is physiological, as it restores hepatopetal flow by redirecting splanchnic portal flow back towards the liver. This minimizes liver dysfunction, reduces protein C, protein S and antithrombin III deficiency, and reduces the risk of hepatic encephalopathy [3]. However, this comes with risks of nerve injury and prominent neck scar(s) from neck dissection. The technique has since evolved. Modified Rex shunts employ different conduits as well as venous inflows, and can be broadly classified into spleno-portal shunts [6,7], gastro-portal shunts [8,9], and porto-portal shunts [3,10]. Other than using the LPV as the venous outlet, bypass from the main portal vein (MPV) to the right portal vein using an inferior mesenteric vein (IMV) or ileal mesenteric vein autograft has also been described in cases of failed Rex shunts [10].

The most suitable technique depends on the availability and accessibility of the inflow vein, outflow vein, and conduit. We report our experience with a portal-Rex shunt using the patent proximal MPV instead of the SMV as the inflow vein for a 9-year-old girl with portal hypertension and distal MPV cavernoma from portal vein thrombosis.

## 2. Case Summary

A 9-year-old girl with unremarkable perinatal history and under follow-up overseas for idiopathic thrombocytopenia was referred to us for consideration of meso-Rex shunt to treat EHPVO. Her thrombocytopenia was incidentally detected during workup for viral infection at 2 years old. Clinically, she had intermittent abdominal pain, as well as portal hypertension with asymptomatic varices and hypersplenism (Hemoglobin 11.5 g/dL, white blood cell count 2.23 × 10^9^ /L, platelet count 48 × 10^9^ /L). Lupus, genetic, and thrombocytopenia workups were negative. Her liver function was normal. Previously, a computed tomography (CT) scan had reported dilated cavernous transformation of the portal vein at the hilum with multiple gastric and splenic varices, moderate splenomegaly, and a small spontaneous splenorenal shunt. The liver was smooth with no focal lesions or cirrhosis. There was no intrahepatic biliary dilatation.

Liver ultrasound scans at our institution revealed a thrombus at the MPV and worsening splenomegaly (15.4 cm). Thrombophilia workup revealed borderline low protein C (72–73%), which was unable to account for the thrombosis in the portal vein. Metabolic, autoimmune and hepatitis workups were negative. Following multidisciplinary discussion with gastroenterologists, pediatric surgeons, and radiologists at our institution, a meso-Rex shunt was recommended. A preoperative CT scan further visualized a tortuous portal vein with cavernous transformation at the hilum and a patent LPV. The liver had remained smooth without focal lesions. Varices were seen around the lesser curve of the stomach and the distal esophagus. The intrahepatic portal vein was not clearly delineated on the CT scan. Hence, a MeVis^®^ (Frauenhofer MeVis Liver Suite, Lübeck, Germany) reconstruction of the CT images was obtained. This revealed patent intrahepatic portal veins. Thus, a transjugular portal venogram was performed in preparation for a meso-Rex shunt. The LPV was patent, and the wedged hepatic venous pressure was 14. Transjugular liver biopsy showed no liver pathology. Preoperative ultrasounds visualized bilateral patent IJVs of good calibre. The SMV was patent with preserved Doppler flow. The splenic vein was mildly dilated and tortuous.

**Figure 1:**
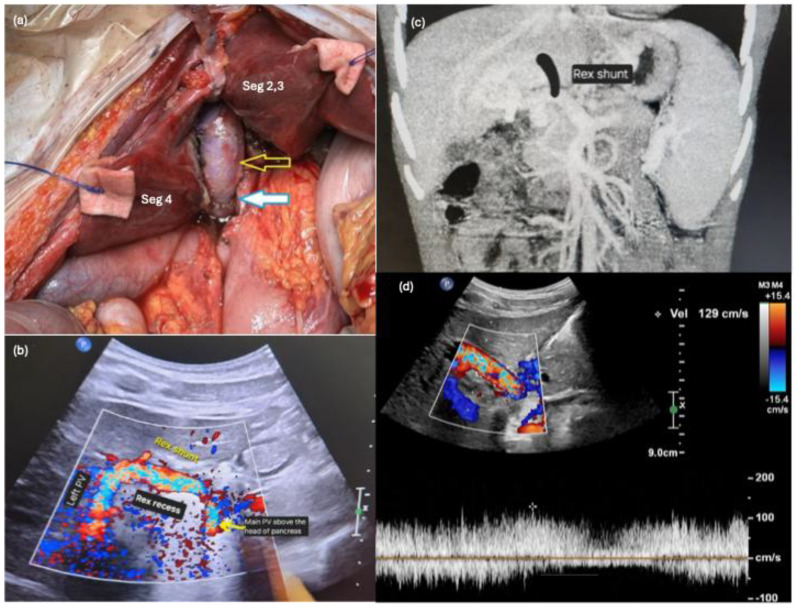
Portal-Rex shunt for extrahepatic portal vein obstruction. (**a**) Intraoperatively, a wedge of liver tissue was resected on either side of the Rex recess for better exposure and to prevent future shunt compression. The left internal jugular vein conduit was anastomosed to the intrahepatic left portal vein and the extrahepatic main portal vein above the head of the pancreas. (Top, hollow arrow—IJV conduit in the Rex recess, bottom solid arrow—IJV-portal vein anastomosis.) (**b**) Post-anastomosis, there was good Doppler signal on intraoperative ultrasound. (**c**) Position of the Rex shunt was annotated on the coronal view of her abdominal computed tomography scan. (**d**) Good hepatopetal flow (129 cm/s) was demonstrated across the portal-Rex shunt 1 year post-operation.

The girl underwent abdominal exploration with a view to perform either a meso-Rex or a modified meso-Rex shunt as per the operative findings below. After entering the abdomen with an upper midline incision, the liver was mobilized to the Rex recess. A bridge of liver tissue covering the Rex recess was opened, and a wedge of liver tissue was resected on either side of the Rex recess to optimize exposure and prevent future shunt compression within the Rex recess (Figure 1a).

Cavernous transformation of the portal vein was found to be limited to the distal MPV. The proximal MPV at the confluence of the SMV and splenic vein was patent, non-sclerotic, not cavernous, and was without internal thrombus. Hence, this part of the portal vein was dissected free and seemed amenable to be utilized to implant the interposition vein graft. No thrombus was found within the distal MPV. A mesenteric vein was cannulated with the catheter tip at the spleno-portal confluence for intraoperative measurements of the portal pressures. No suitable conduit was identified intra-abdominally as the veins were of poor quality, and the left gonadal and inferior mesenteric veins were small in calibre. Thus, left neck exploration was performed via two transverse neck incisions (left infra-mastoid and left supraclavicular) to harvest the left IJV as a conduit (4 cm long, 9 mm in diameter). The extracted tissue adjacent to the harvested left IJV was unfortunately suspicious for a nerve. The graft was flushed with heparinized saline and anastomosed to the LPV at the Rex recess, followed by the proximal MPV in an end-to-side manner (Figure 1a). There was prompt reduction in the portal pressure from 36 mmHg before to 7 mmHg after shunt creation. Post-anastomosis Doppler ultrasound of the liver confirmed good flow within the left portal vein (Flow velocity 20–50 cm/s) (Figure 1b). Figure 1c illustrates the position of the Rex shunt superimposed on the coronal view of the patient’s abdominal CT scan.

Ultrasound surveillance was performed daily from postoperative days 1 to 5. There was good hepatopetal flow in the interposition vein graft (218 > 223 > 187 > 202 > 198 cm/s) and the LPV (72.5 > 48 > 45 > 67 > 64.2 cm/s). Prophylactic heparinization was given from postoperative day 1 (IV heparin 10 u/kg/h) and bridged with subcutaneous enoxaparin (30 mg OD) from postoperative day 5 to her first outpatient review in view of transaminitis. She was discharged on postoperative day 6. Postoperative transient transaminitis resolved at the first outpatient review with her hematology specialist on postoperative day 17, and subcutaneous enoxaparin was thus converted to rivaroxaban (10 mg OD) to complete 3 months of prophylactic anticoagulation against Rex shunt thrombosis. She was followed up with serial ultrasounds at 3 weeks, 6 weeks, 3 months, 6 months, 9 months and 12 months post-operation. Throughout the clinical follow-up period, ultrasound scans showed a patent shunt with good hepatopetal flow (129 cm/s at 1 year post-operation) (Figure 1d) and improvement in splenomegaly (from 15.4 cm before surgery to 12.1 cm 1 year post-operation). Her thrombocytopenia also improved (from a platelet count of 46 × 10^9^ /L pre-operation to 123 × 10^9^ /L at 1 year post-operation). Ultrasound scan at 3 weeks post-operation initially detected a narrowing near the IJV–MPV anastomotic site reportedly due to postoperative edema, which improved from a luminal diameter of 1.9 mm (3 weeks post-operation) to 4.5 mm (1 year post-operation). She sustained left vocal cord palsy from iatrogenic left vagus nerve injury, and was treated with injection medialization of the left vocal cord by Otorhinolaryngology and speech therapy with subsequent resolution of hoarseness of voice.

## 3. Discussion

Selective Rex shunts are preferred over central portosystemic shunts as the mainstay surgical treatment of EHPVO in children [3,11]. The Rex shunt is advantageous due to its ability to achieve both portal decompression and augmentation of portal flow in a hepatopetal direction, thus avoiding liver dysfunction, hepatic encephalopathy, and liver decompensation [3]. Modified Rex shunts provide the versatility of tailoring this bypass to the patient’s anatomy, with similar shunt success as a meso-Rex bypass. We report a successful portal-Rex shunt in a 9-year-old girl using IJV as conduit and proximal MPV as the venous inlet.

Unlike the other portal–portal shunts in the literature, we chose to use IJV instead of an intra-abdominal vein because of the lack of suitable intra-abdominal veins of sufficient calibre and quality [3,12]. Our first choice of conduit was an intra-abdominal vein such as the IMV of adequate calibre (8–10 mm), as this avoids the neck dissection and the potential morbidity associated with IJV harvesting [13,14,15]. However, there were no suitable intra-abdominal veins available on intraoperative inspection. Despite the associated morbidity of neck dissection, the IJV was a superior choice as her IJV was of good calibre (9 mm), had no collateral branches, and was not tortuous, albeit with the risk associated with neck exploration [3]. Furthermore, the IJV conduit has been shown to have a better patency rate compared to the intra-abdominal veins [16]. Alternative conduits for Rex shunts described in the literature include the great saphenous vein (GSV), prosthetic grafts, splenic, coronary, IMV and umbilical veins. However, harvesting the GSV requires a separate scar, and prosthetic grafts carry higher thrombotic and infection risks [4]. Thus, the IJV was selected as the preferred conduit in her case.

Intraoperative close inspection of the extrahepatic portal anatomy revealed that the cavernous transformation had only affected the distal MPV, leaving sufficient length of good-quality proximal MPV at the portal vein–SMV–splenic vein confluence to anastomose to the IJV conduit. The advantage in anastomosing the conduit to the MPV is a readily available inflow, as well as a shorter and more direct conduit. Shorter and direct venous conduits may be less prone to kinks, twists and thrombosis [17]. Zhang et al. reported successful implementation of the portal cavernoma-Rex shunt with an interposition of portal vessels such as the jejunum, ileal or inferior mesenteric vein in 18 children [12]. The same authors 2 years later reported eight cases of occlusion and narrowing of the portal cavernoma–left portal bypass requiring a second Rex shunt [18]. We avoided using the diseased cavernous portion of the portal vein as the anastomotic site to optimize the portal vein quality at the anastomosis and improve flow dynamics at the junction.

Intraoperative ultrasound Doppler and pressure measurements at the spleno-portal confluence following shunt creation confirmed patency, good blood flow and successful portal decompression with reduction in portal pressure. The shunt was closely monitored on ultrasound for early and late complications such as thrombosis and stenosis. On ultrasound, the initial narrowing near the IJV–MPV anastomosis was presumably due to postoperative edema rather than a true anastomotic stenosis, as the luminal diameter improved from 1.9 mm to nearly 4 mm at 1 month post-operation. While the wasting continues to be visible on ultrasound 1 year after operation (4.5 mm), the portal-Rex shunt remains patent with good flow (129 cm/s 1 year post-operation from 218 cm/s immediately post-operation). There was an improvement in platelet count and a reduction in splenic size, suggesting effective restoration of intrahepatic perfusion and relief of portal hypertension.

Patients with EHPVO from portal vein thrombosis may benefit from a comprehensive workup for pro-thrombotic disorders to evaluate for predisposition to graft thrombosis. Our patient had thrombocytopenia and portal vein thrombosis. Underlying hepatic conditions such as hepatitis B/C, alpha-1-antitrypsin deficiency and autoimmune hepatitis were excluded. The malignancy screen was negative. In light of a recent comprehensive review highlighting the importance of antiphospholipid antibodies in vascular disorders of the liver, it may be desirable to investigate for the presence of antiphospholipid syndrome for a heightened surveillance of thrombosis and careful management of postoperative anticoagulation [19].

In conclusion, we report a case of EHPVO with portal decompression and improvement in hypersplenism from a portal-Rex shunt using the IJV as conduit and a patent proximal MPV of good quality as the shunt’s venous inlet. This is a feasible and effective alternative to the classic meso-Rex shunt.

## Data Availability

Data sharing is not applicable as no separate datasets were generated and/or analyzed for this study.

## Abbreviations

The following abbreviations are used in this manuscript:

EHPVO	Extrahepatic portal vein obstruction
SMV	Superior mesenteric vein
IMV	Inferior mesenteric vein
GSV	Great saphenous vein
LPV	Left portal vein
MPV	Main portal vein
IJV	Internal jugular vein
CT	Computed tomography
